# Modeling Climate Refugia for *Chengiodendron marginatum*: Insights for Future Conservation Planning

**DOI:** 10.3390/plants14131961

**Published:** 2025-06-26

**Authors:** Zhirun Yu, Quanhong Yan, Yilin Li, Zheng Yan, Chenlong Fu, Bo Jiang, Lin Chen

**Affiliations:** 1 Co-Innovation Center for the Sustainable Forestry in Southern China, College of Life Sciences, Nanjing Forestry University, Nanjing 210037, China; yuzhirunyu@163.com (Z.Y.); quanhongyan@njfu.edu.cn (Q.Y.); linyili@njfu.edu.cn (Y.L.); yanzheng611@outlook.com (Z.Y.); chenlongfu1219@njfu.edu.cn (C.F.); syouha941211@gmail.com (B.J.); 2 International Cultivar Registration Center for Osmanthus, Nanjing Forestry University, Nanjing 210037, China

**Keywords:** *Chengiodendron marginatum*, climate change, suitable habitats, potential distribution, Maxent model

## Abstract

*Chengiodendron marginatum*, an evergreen tree or shrub belonging to the Oleaceae family, represents a critical germplasm resource with considerable potential for novel cultivar breeding. To elucidate the adaptive responses of *C. marginatum* to climate change and provide strategic guidance for its conservation, this study investigates the changing patterns in its potential suitable habitats under various climate scenarios. We employed an integrated approach combining maximum entropy (Maxent) modeling with GIS spatial analysis, utilizing current occurrence records and paleoclimatic data spanning from the mid-Holocene to future projections (2041–2060 [2050s] and 2061–2080 [2070s]). Climate scenarios SSP126 and SSP585 were selected to represent contrasting emission pathways. The model demonstrated excellent predictive accuracy with an AUC value of 0.942, identifying precipitation-related variables (particularly the precipitation of driest month and annual precipitation) as the primary environmental factors shaping the geographical distribution of *C. marginatum*. Current suitable habitats encompass approximately 98.38 × 10^4^ km^2^, primarily located in East, Central, and South China, with high-suitability habitats restricted to southern Hainan, Taiwan, and northeastern Guangxi. Since the mid-Holocene, an expansion of suitable habitats occurred despite localized contractions in Southwest China. Future projections revealed moderate habitat reduction under both scenarios, and high-suitability areas decreased substantially. Importantly, under both scenarios, persistent high-suitability habitats were maintained in southern Hainan, Taiwan, and northeastern Guangxi, which are identified as essential climate refugia for the species. These findings provide a basis for understanding the response of the species to climate change and offer valuable guidance for its conservation.

## 1. Introduction

Climate is one of the most important factors that determines the suitability of species habitats. Climate change poses serious challenges to biodiversity, significantly impacts species distribution, and may even lead to species extinction [1,2]. The advancement of human industrialization and the development of various production activities have significantly influenced the climate. The global temperature has increased by roughly 0.8 °C in the last century. In the 21st century, the warming trend is predicted to continue [3,4]. For plants, rising temperatures disrupt growth patterns, physiological functions, and historical habitat suitability, contributing to accelerated biodiversity loss [5]. Therefore, comprehending the impact of future climate change on plants and predicting the potential habitats of plants are essential for developing appropriate species conservation strategies and measures [6]. In this context, species distribution models (SDMs) provide a critical framework to predict climate-driven habitat changes and inform conservation strategies.

For the purpose of developing successful biodiversity conservation plans and evaluating the effects of climate change on species distribution, it is crucial to investigate the global distribution patterns of species and forecast their prospective geographic distribution [7]. Recently, SDMs have been widely used to forecast the current distribution pattern of species and evaluate the effects of climate change on species distribution [8,9]. SDMs statistically link species occurrence data to environmental variables (e.g., temperature, precipitation) to assess habitat suitability and predict geographic distributions. By quantifying species–environment relationships, SDMs forecast range shifts and identify suitable areas under different scenarios [10,11,12].

By using SDMs to predict the potential habitat of species, it is possible to develop corresponding ecological restoration and species protection strategies, which improves species protection efforts more precisely [6,13]. Currently, GARP, Bioclim, DOMAIN, and Maxent are the most widely used models for predicting species distribution [12,14,15,16]. The Maxent model is based on the maximum entropy theory and describes the probability distribution of a set of environmental grids and geographically referenced occurrence locations, where each grid cell predicts the suitable conditions for the species [17,18]. Compared to other models like Bioclim and GARP, Maxent demonstrates higher flexibility and accuracy, particularly under limited occurrence data conditions [19,20]. The Maxent model can be used not only for the prediction and analysis of widely distributed species [21,22,23] but also for narrowly distributed or endangered species [6,24,25].

The genus *Chengiodendron* belongs to the family Oleaceae and was originally a panicle group (sect. *Leiolea*) of the genus *Osmanthus*. The phylogenetic relationships of four chloroplast gene sequences (*trnL-F*, *trnT-L*, *trns-G*, and *matK*) and nuclear gene fragments (ITS) indicated that *Chengiodendron* is a monophyletic group independent of *Osmanthus* [26]. *C. marginatum* is an evergreen shrub or tree with leathery leaves and grows in valleys and dense forests on hillsides at an altitude of 800–1800 m. It is mainly distributed in East, Central, South, and Southwest China [27]. Despite its ecological uniqueness and value as a germplasm resource for *Osmanthus* breeding, wild populations of *C. marginatum* are fragmented and declining due to human-induced disturbances, which require urgent conservation measures. However, relevant research has mainly focused on a few species like *Osmanthus fragrans* [28,29], and there is a lack of research on *C. marginatum*. Therefore, clarifying the geographical distribution of this species is of great significance for the protection and utilization of the germplasm resources of this species. This study aims to (a) predict the current and future potential distribution of *C. marginatum* across historical, contemporary, and future climate scenarios using the Maxent model and GIS and (b) identify the most significant climatic factors driving its habitat suitability. These objectives address critical knowledge gaps by delivering the first comprehensive SDM assessment for *C. marginatum*—an ecologically unique Oleaceae species—through an integrated framework combining paleoclimatic, contemporary, and future projections to identify climate refugia. The findings provide critical insights for understanding the species’ response to climate change and inform conservation strategies for its germplasm resources.

## 2. Results

### 2.1. Accuracy of Maximum Entropy Model Detection

**Figure 1:**
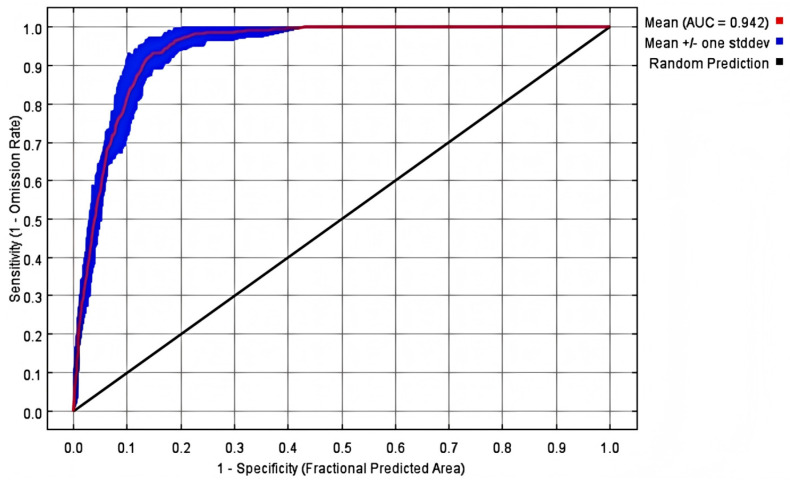
The ROC curve test of the prediction results of *C. marginatum*.

The simulation results of the Maxent model based on 248 valid records and 24 environmental variables showed that the AUC value of training data was relatively high, indicating that the model had good predictive ability and applicability (Figure 1).

### 2.2. Analysis of Environmental Variables Influencing Distribution of C. marginatum

**Figure A1:**
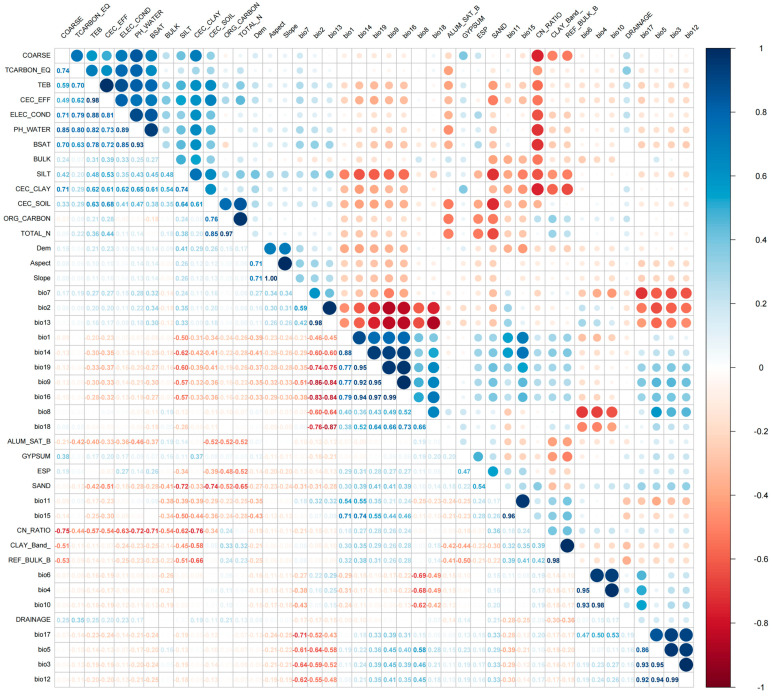
Correlation analysis of geographical distribution and environmental variables of *C. marginatum.* Circle sizes indicate the magnitude of pairwise correlation coefficients between variables.

Based on the robust predictive accuracy of the model, we next identify the key environmental drivers shaping the distribution of *C. marginatum*. According to the correlation analysis results (Figure A1), 24 variables were retained for subsequent analyses, including eight bioclimatic, fourteen soil, and two topographic variables (Table 1).

**Figure 2:**
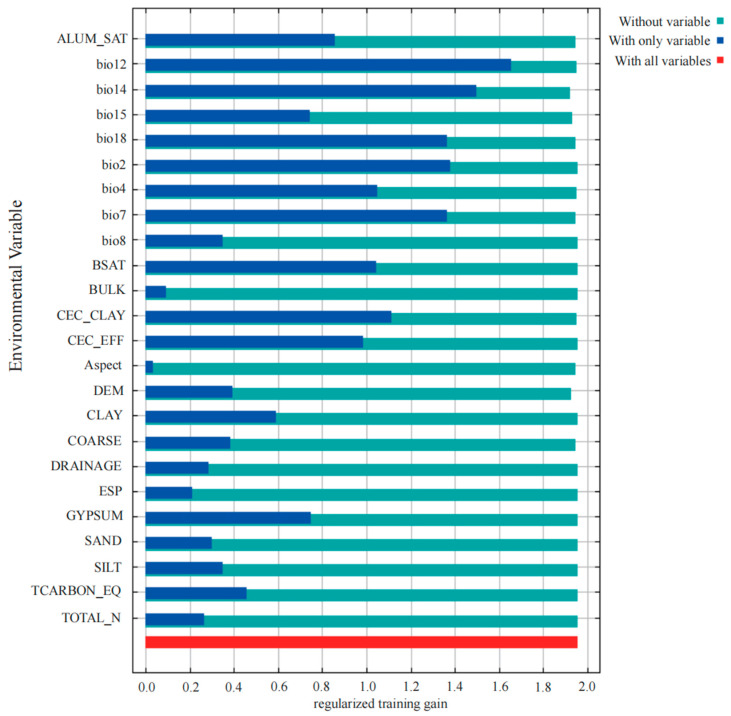
Jackknife test result of environmental factors for *C. marginatum*.

According to the percentage contribution and permutation importance results (Table 1), the top eight environmental factors influencing the distribution of *C. marginatum* were the precipitation of driest month (bio14, 58.3%), annual precipitation (bio12, 14.9%), temperature annual range (bio7, 5.5%), aluminum saturation (ALUM_SAT, 4.9%), elevation (DEM, 2.4%), temperature seasonality (bio4, 2.5%), clay cation exchange capacity (CEC_CLAY, 1.9%), and base saturation (BSAT, 2.9%). Among these, bio14 and bio12 were the dominant factors, jointly accounting for 73.2% of the total contribution (Table 1). When using only individual variables for model prediction, bio12 had the highest regularized training gain, followed by bio14. The Jackknife test further indicated that bio7, bio2 (mean diurnal range), and bio18 (precipitation of warmest quarter) also exhibited relatively high training gains, though the differences among them were marginal (Figure 2).

The jackknife test, permutation importance, and percentage contribution indicated that the precipitation of the driest month and annual precipitation were important factors affecting the distribution of potential suitable areas of *C. marginatum*, with a stronger influence than both temperature and soil variables.

**Figure 3:**
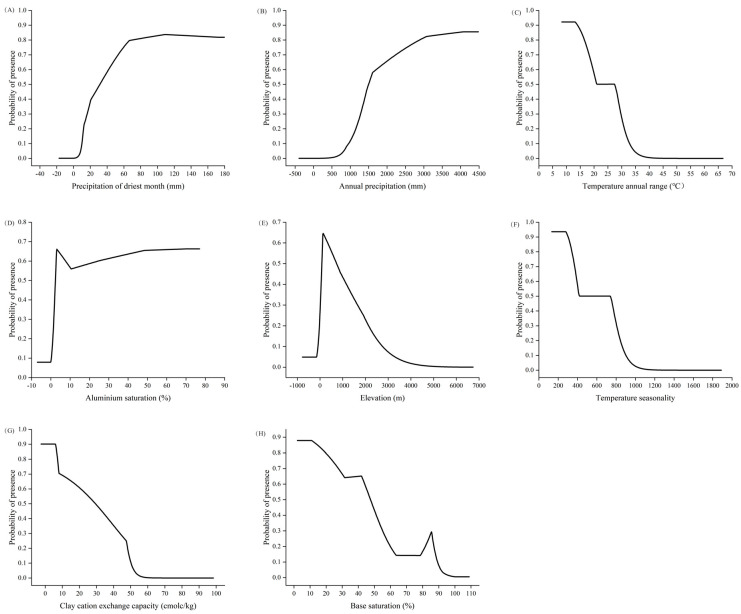
Response curves of the existence probability of *C. marginatum* for environmental variables: (**A**) bio14, (**B**) bio12, (**C**) bio7, (**D**) ALUM_SAT, (**E**) DEM, (**F**) bio4, (**G**) CEC_CLAY, (**H**) BSAT.

Based on the response curves of each environmental factor (Figure 3), the optimal habitat conditions for *C. marginatum* were a driest monthly precipitation of 31.51 mm–179.71 mm, an average annual precipitation of 1497.43 mm–4481.30 mm, a range of annual temperature variations of 8.32 °C–27.36 °C, an aluminum saturation of 2.39–76.67%, an elevation of 81.43 m–727.67 m, a temperature seasonality of 133.56–737.35, a clay cation exchange capacity of 0.90 cmolc/kg–29.45 cmolc/kg, and a base saturation of 2.1–47.38%.

### 2.3. Distribution of Potential Suitable Habitats of C. marginatum

**Figure 4:**
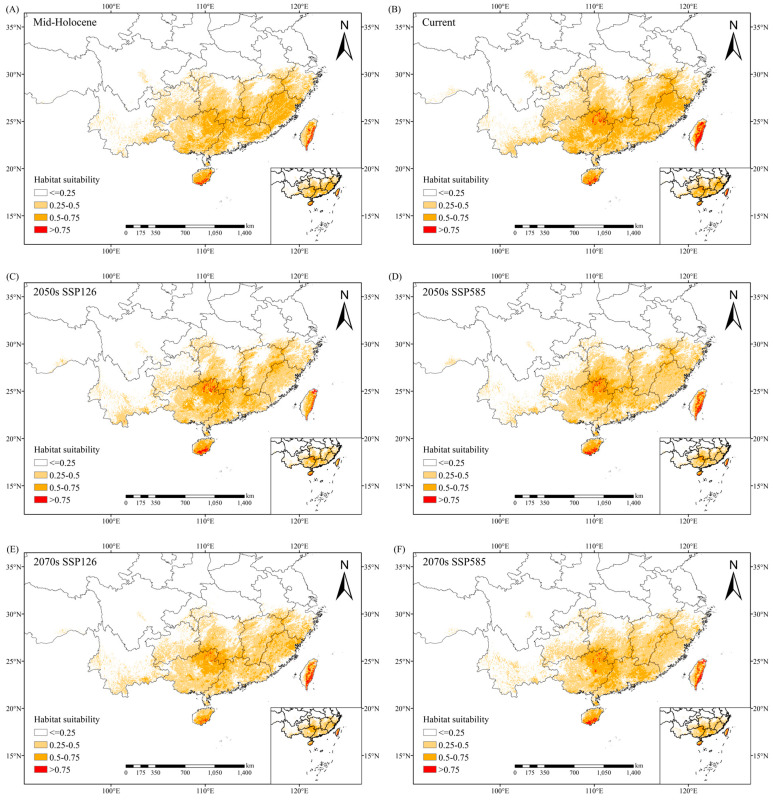
Distribution of potential suitable areas for *C. marginatum* under different climate scenarios: (**A**) mid-Holocene, (**B**) current, (**C**) 2050s SSP126, (**D**) 2050s SSP585, (**E**) 2070s SSP126, (**F**) 2070s SSP585.

The potential distribution of *C. marginatum* under contemporary climate conditions is shown in Figure 4. The high-, medium-, and low-suitability habitats were 1.62 × 10^4^ km^2^, 31.04 × 10^4^ km^2^, and 65.72 × 10^4^ km^2^, respectively, with a total area of 98.38 × 10^4^ km^2^ (Table 2), accounting for approximately 10.25% of China’s total land area. According to the results presented by the Maxent model, the current high-suitability habitat was mainly distributed in the southern part of Hainan Island and the central and eastern part of Taiwan Island, with scattered high-suitability areas also identified in northeastern Guangxi. The medium-suitability habitat covered southern Zhejiang, most of Fujian, eastern Jiangxi, southern Hunan, most of Guangdong and Guangxi, and Hainan Island and Taiwan Island. The low-suitability habitat mainly included Hunan, most of Jiangxi and Zhejiang, southwestern Hubei, Yunnan, Guizhou, southern Anhui, and parts of Guangdong, Guangxi, and Fujian, with a small amount distributed in Tibet, Sichuan, Chongqing, and Taiwan.

**Figure 5:**
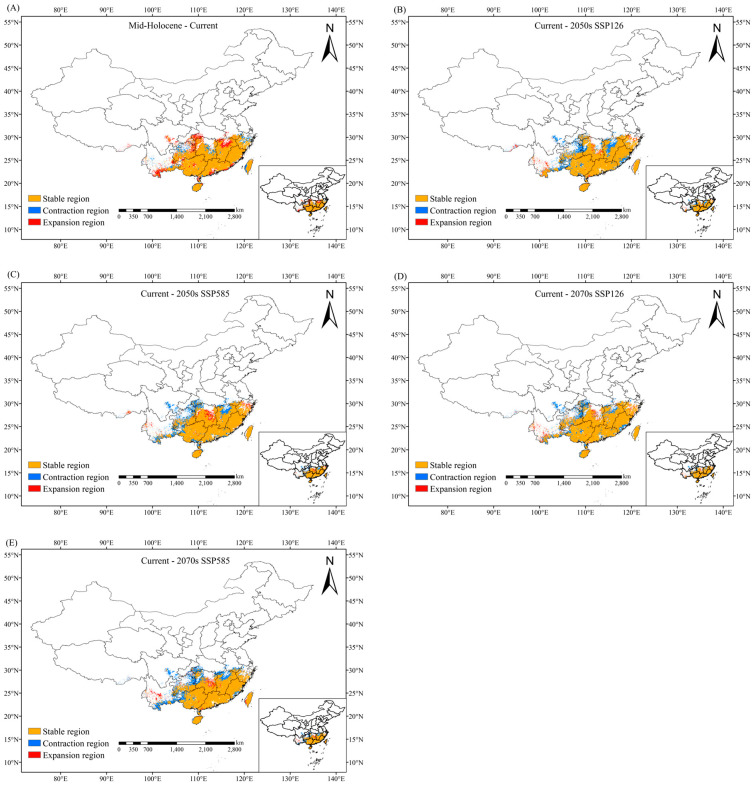
Changes in suitable habitat of *C. marginatum* under different climate scenarios compared to contemporary conditions: (**A**) mid-Holocene-current, (**B**) current-2050s SSP126, (**C**) current-2050s SSP585, (**D**) current-2070s SSP126, (**E**) current-2070s SSP585. (Red, orange, and blue represent expansion, stability, and contraction regions, respectively.)

In the middle Holocene, the total suitable habitat area was 89.37 × 10^4^ km^2^. From the mid-Holocene to the present, the species’ suitable habitat expanded significantly in Yunnan, northern Jiangxi, and southwestern Hubei; the border regions of Hubei, Hunan, Guizhou, and Chongqing; and there were additional expansions observed in localized areas of Sichuan, Guangxi, Guangdong, and Fujian. Minor contractions were interspersed in parts of Zhejiang, Taiwan, central–eastern Hunan, and Yunnan, though these reductions did not alter the overall expansion trend (Figure 5). High-suitability habitats expanded notably in Taiwan and northeastern Guangxi relative to the mid-Holocene (Figure 4).

Projections under different SSP scenarios reveal distinct development pathways—sustainable development (SSP126) and fossil-fueled development (SSP585)—with habitat shifts analyzed across different periods and climate scenarios. Under the SSP126 future climate scenario, the total suitable habitat area of *C. marginatum* during 2041–2060 (2050s) is projected to decrease by 14.33% compared to contemporary conditions, with notable contractions observed in Yunnan, central Jiangxi, southeastern Fujian, and the border regions of Hubei, Hunan, Guizhou, and Chongqing. Moderate reductions also occurred in parts of Sichuan and Guangxi, while limited expansions were detected in isolated areas of Hunan, Zhejiang, and western Yunnan (Figure 5, Table 2). The area of high-suitability habitats decreased by 35.19% compared to contemporary conditions, with Taiwan experiencing substantial contraction (Figure 4, Table 2). The total suitable habitat area in 2061–2080 (2070s) was 86.37 × 10^4^ km^2^, which was 12.21% smaller than the current climate scenario. Significant contractions are predicted in Yunnan, northern Jiangxi, southeastern Fujian, southwestern Hubei, and the border regions of Hubei, Hunan, Guizhou, and Chongqing, with moderate declines extending to parts of Sichuan and Guangxi. Limited expansions are restricted to scattered areas of Hunan, Zhejiang, and western Yunnan (Figure 5, Table 2). The area of high-suitability habitats decreases by 44.44% (Figure 4, Table 2).

Under the climate scenario of SSP585, the total suitable habitat area in 2041–2060 was 86.01 × 10^4^ km^2^, a decrease of 12.57% compared with the contemporary climate scenario. Major expansions are projected in central–eastern Hunan, alongside localized habitat gains in parts of northern Zhejiang and Yunnan. In contrast, substantial contractions are anticipated in southwestern Hubei and the border regions of Hubei, Hunan, Guizhou, and Chongqing, as well as northern Jiangxi, with minor reductions observed in Sichuan, Yunnan, and the Guangdong–Guangxi regions (Figure 5, Table 2). High-suitability habitats showed a contraction of 19.75% in area under this scenario compared to contemporary conditions (Figure 4, Table 2). The total suitable habitat area from 2061 to 2080 was 82.18 × 10^4^ km^2^, which was 16.47% smaller than that in the contemporary climate scenario. Relatively obvious contractions are observed in Yunnan, northern Jiangxi, southern Anhui, southeastern Fujian, southwestern Hubei, and the border regions of Hubei, Hunan, Guizhou, and Chongqing, with additional reductions in parts of Sichuan and Guangxi. Conversely, moderate expansions are limited to scattered areas of eastern Hunan and central Yunnan (Figure 5, Table 2). The total area of highly suitable areas decreased by 30.25% compared with the contemporary climate (Figure 4, Table 2).

### 2.4. Migration of Suitable Habitat Center

**Figure 6:**
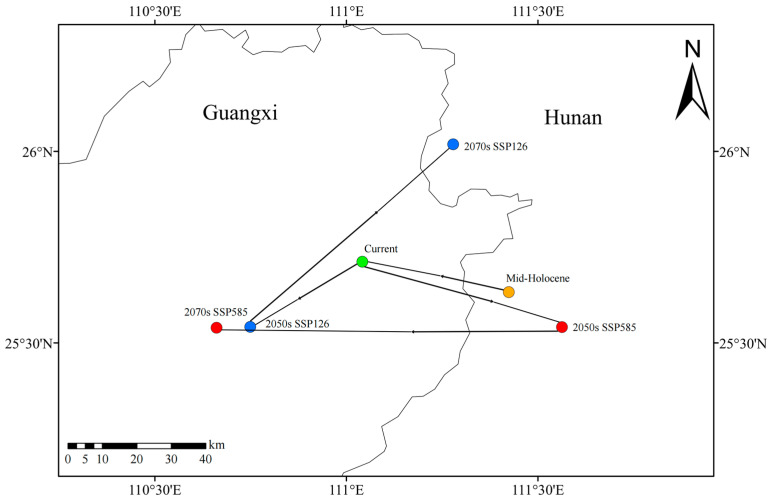
Shift in suitable habitat distribution center in climate scenarios.

The centroid of *C. marginatum*’s suitable habitats shifted from Yongzhou City, Hunan Province (111.42° E, 25.63° N), during the mid-Holocene to Guilin City, Guangxi Zhuang Autonomous Region (111.04° E, 25.71° N), under contemporary conditions. Under the SSP126 scenario, the centroid is projected to remain in Guilin, Guangxi (110.75° E, 25.54° N), by the 2050s but shifts northeastward back to Yongzhou, Hunan (111.28° E, 26.02° N), by the 2070s. Under the SSP585 scenario, the centroid migrates southeastward to Yongzhou, Hunan (111.56° E, 25.54° N), by the 2050s then shifts westward to Guilin, Guangxi (110.66° E, 25.54° N), by the 2070s (Figure 6).

## 3. Discussion

### 3.1. Model Evaluation and Limitations

The Maxent model identified precipitation of the driest month (bio14) and annual precipitation (bio12) as the dominant drivers of *C. marginatum* distribution, jointly accounting for 73.2% of the total variable contribution—significantly exceeding the combined contributions of temperature variables, soil properties, and topographic factors (elevation, slope, aspect). High predictive accuracy was confirmed (AUC = 0.942) with a sample size ≥5 exceeding reliability thresholds [30]. The species distribution data we used come from multiple databases, and there was also distribution data obtained through field surveys. To minimize overfitting and enhance model performance, we correlated species distribution data with environmental variables, retaining only one instance of redundant environmental raster data [31].

While this study incorporated multi-dimensional environmental predictors, anthropogenic factors like habitat fragmentation were not explicitly quantified. For example, the coastal regions of Fujian and Zhejiang show high predicted suitability in our model (Figure 4B) but limited actual occurrences, likely due to intensive land development causing habitat fragmentation. Consequently, discrepancies between predicted suitable habitats and actual species distributions may persist, particularly in human-dominated landscapes. Future work should integrate socio-ecological variables to refine model realism and support conservation planning.

### 3.2. Key Environmental Factors Influencing C. marginatum Distribution

Temperature and precipitation are two key climate factors that influence species distribution and the features of forest ecosystems [32,33]. According to this study, the most significant environmental factors affecting the geographical distribution of *C. marginatum* were precipitation of the driest month and annual precipitation. The environmental factors related to precipitation have a significant impact on the distribution of species, which is similar to the results of *Osmanthus fragrans* [34]. *Castanopsis carlesii* is widely distributed across regions south of the Yangtze River, sharing a similar range with *C. marginatum* but exhibiting greater sensitivity to dry season precipitation. Research has shown that precipitation is the primary environmental element influencing *Castanopsis carlesii* distribution, with precipitation in the driest month playing a particularly critical role [35]. These findings are essentially consistent with our study. At present, the distribution of *C. marginatum* is mainly concentrated in the subtropical region south of the Yangtze River Basin, and the suitability generally decreases from the southeast coast to the northwest inland, which is similar to the overall distribution characteristics of annual precipitation in China. In this study, temperature had less of an impact on the distribution of *C. marginatum* than precipitation factors did. However, temperature does play an important role in plant growth and reproduction. For instance, accumulated temperature is a key factor in the growth of *Cinnamomum camphora* [36], and low temperature can limit the geographical distribution of *Quercus fabri* [37], suggesting that the effects of climate factors on plant distribution may be species-specific. While soil properties and topographic variables were incorporated into the updated model, their combined contributions remained secondary (contributing <10%), further highlighting the dominant influence of moisture availability in shaping habitat suitability for *C. marginatum*.

### 3.3. Changes in Suitable Habitat of C. marginatum Under Different Climate Scenarios

The phenomenon of global climate change will result in alterations to the original temperature and precipitation patterns [38]. The species will progressively move to a new environment that is more suitable for survival as a result of changes in the initial habitat circumstances, which will alter the species’ distribution region [39]. Studies have shown that in terms of global warming, many species will migrate to high-latitude and high-altitude regions [40]. However, some species can adjust to shifting climatic conditions, and the range of suitable habitats for these species are continuously growing [41]. To assess *C. marginatum*’s response to global climate change, we evaluated habitat dynamics under future scenarios. We found that the changes in the suitable habitat of *C. marginatum* varied in different periods and under different climate scenarios.

From the mid-Holocene to present, the suitable habitats exhibited significant habitat expansion in northern Jiangxi, southwestern Hubei, and the border areas of Hubei, Hunan, Guizhou, and Chongqing (Figure 5). This trend coincides with late Holocene moisture recovery in the mid-Yangtze region [42], which likely compensated for regional dry-season precipitation declines. From the perspective of the future habitable range, there will be an expansion trend in the southwest of China, which may be related to the higher altitude in the southwest of China.

Under the SSP126 scenario, habitat contraction occurred in both periods, where the total suitable area decreased by 14.33% (2050s) and 12.21% (2070s) relative to contemporary conditions. Losses were concentrated in Yunnan, Jiangxi (central and northern), southeastern Fujian, and the Hubei–Hunan–Guizhou–Chongqing borders, with moderate declines in Sichuan and Guangxi. Concurrently, high-suitability areas contracted substantially (35.19–44.44%), particularly in Taiwan. Habitat loss progressed under the SSP585 scenario, increasing from 12.57% (2050s) to 16.47% (2070s), with significant reductions concentrated in the Hubei–Hunan–Guizhou–Chongqing border region, as well as northern Jiangxi. Despite this, high-suitability habitats persisted in northeastern Guangxi, Taiwan, and southern Hainan across all scenarios. Geographic areas that are less affected by climate change and maintain relatively stable habitats can provide ideal natural environments for the survival and reproduction of organisms, and overlapping areas of current and future suitable distribution can be designated as refugia [43]. According to the above research results, *C. marginatum* may have refugia in northeastern Guangxi, Taiwan, and the southern part of Hainan Island in the future.

Centroid migration patterns exhibit scenario-dependent trajectories. Under SSP126, the centroid stabilizes in Guilin, Guangxi (110.75° E, 25.54° N), during the 2050s before shifting northeastward to Yongzhou, Hunan (111.28° E, 26.02° N), by the 2070s. Under SSP585, a distinct southeastward migration occurs to Yongzhou, Hunan (111.56° E, 25.54° N), in the 2050s, followed by westward movement to Guilin, Guangxi (110.66° E, 25.54° N), in the 2070s. Both scenarios ultimately converge in northeastern Guangxi, confirming its status as a persistent climate refugia.

### 3.4. Conservation Strategy of C. marginatum

*C. marginatum,* a species with ecological significance, scientific research value [26], and potential for economic utilization, faces severe challenges due to human-induced disturbances (e.g., land development and resource overexploitation). The habitat fragmentation of its wild populations is intensifying, and the current resource base is under significant threat. Effective conservation must integrate scientific research with proactive management strategies, including habitat protection, restoration, and ex situ conservation efforts.

Our study confirms that southern Hainan, Taiwan, and northeastern Guangxi function as critical climate refugia, maintaining persistent high-suitability habitats under all climatic periods. These regions necessitate urgent prioritization through expanded protected areas and strict development restrictions to ensure population viability. In Yunnan and Guangxi—where future habitat expansion is projected—targeted restoration via assisted migration and native vegetation recovery should enhance landscape connectivity. Central and eastern Chinese provinces, particularly those experiencing severe habitat degradation (e.g., Hubei, Jiangxi, Fujian), require integrated emission mitigation through renewable energy transition and large-scale reforestation initiatives. Complementary efforts should leverage the species’ horticultural value through artificial propagation in confirmed high-suitability zones and ex situ conservation via seed banking, reducing wild population pressures while safeguarding genetic diversity for *Osmanthus* breeding applications.

## 4. Materials and Methods

### 4.1. Species Data Collection

**Figure 7:**
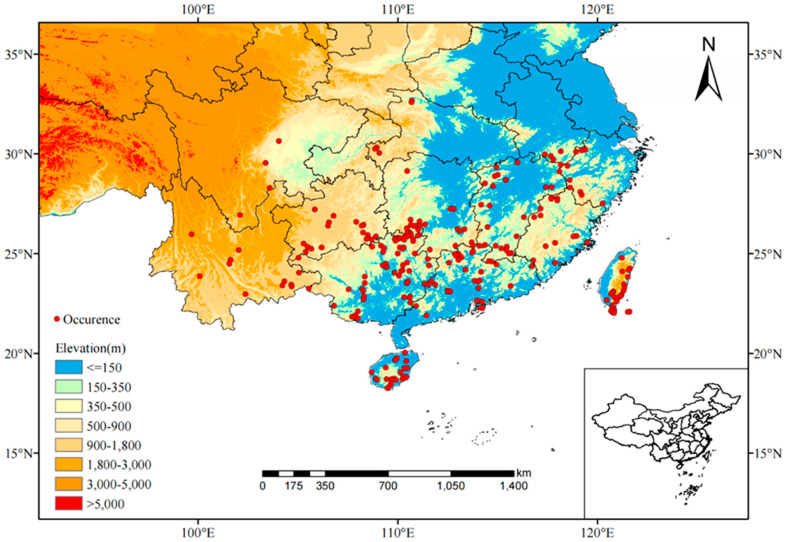
Locations of 248 selected occurrence records (shown by red dots) of *C. marginatum* in China.

The geographical distribution data of *C. marginatum* were obtained through field surveys and database retrieval. The databases include the Global Biodiversity Information Facility (GBIF, https://www.gbif.org), the National Specimen Information Infrastructure (NSII, http://www.nsii.org.cn), and the Chinese Virtual Herbarium (CVH, https://www.cvh.ac.cn/). A total of 517 initial records were obtained. ArcGIS was used to check and screen the distribution points, and duplicate, incomplete, and inaccurate records were deleted. To avoid errors caused by high density, we utilized a grid cell size of 2.5′ for spatial filtering, retaining only one occurrence point per grid cell. Finally, 248 point locations of the species were obtained (Figure 7). The administrative division map of China was downloaded from the National Basic Geographic Information Center (https://www.ngcc.cn/) and then clipped using ArcGIS 10.8 software and converted to ASCII format.

### 4.2. Environment Variable Data Collection

The 19 bioclimatic data points used in this study were obtained from the WorldClim global climate database (https://worldclim.org/). The mid-Holocene data were obtained from WorldClim V1.4 [44]; current data are the average data from 1970 to 2000 in WorldClim V2.1 [45]. The Sixth International Coupled Model Intercomparison Project (CMIP6) provides a standardized framework for global climate projections, integrating atmosphere–ocean general circulation models to simulate future climate conditions under different scenarios. The potential geographical distribution of future climate scenarios is predicted using the Beijing Climate Center Climate System Model (BCC-CSM2-MR model), a CMIP6-endorsed model known for its good predictive capability [46]. The Shared Socioeconomic Pathways (SSPs) are scenarios combining socioeconomic development pathways with greenhouse gas emission levels, widely adopted in CMIP6 to assess climate risks. SSP126 represents a pathway centered on sustainability, characterized by forcing a stabilized climate, driven by low greenhouse gas emissions, renewable energy adoption, and balanced policies regulating land use. In contrast, SSP585 follows a fossil-fueled development path marked by forcing an unmitigated climate increase, reflecting rapid industrialization, high energy demand, and minimal climate mitigation efforts [47,48]. This study selected two scenarios, SSP126 and SSP585, and two periods, 2041–2060 (2050s) and 2061–2080 (2070s), as future environmental data.

The 21 soil variables were derived from HWSD2.0 of the Harmonized World Soil Database (https://www.fao.org/soils-portal/data-hub/soil-maps-and-databases/harmonized-world-soil-database-v20/, accessed on 20 April 2025) [49]. Compared to the previously used HWSD1.2 dataset, HWSD2.0 provides more comprehensive soil parameters. Soil data in this dataset are stratified into seven depth layers (D1-D7). Based on the root distribution characteristics of *Osmanthus* relatives [50] and field investigations of *C. marginatum*, the D2 layer (20–40 cm depth) was selected for analysis. Topographic variables (elevation, slope, and aspect) were sourced from the Shuttle Radar Topography Mission (https://www.jpl.nasa.gov/missions/shuttle-radar-topography-mission-srtm, accessed on 10 January 2025). The specific environmental variables selected for this study can be found in Table A1.

### 4.3. Model Construction and Evaluation

ArcGIS 10.8 software was used to extract the climate data of *C. marginatum*. At the same time, pairwise Pearson correlation analysis was performed among all climate variables using R 4.2.2 and the ‘ggcorrplot’ R package (version: 0.1.4.1) to calculate the correlation coefficient between variables. For variables with pairwise correlation coefficients of |r| ≥ 0.8, those exhibiting higher contributions were retained for model development. The coordinate information in CSV format and the selected environmental variables were imported into Maxent 3.4.4 software, and the operation was repeated 10 times. Using the cross-validation method, all the coordinate information was randomly divided into ten subsets, one of which was used as the validation set and the rest as the training set. The final output was the average result of the ten operations [51]. The acceptance (ROC) curve was drawn with the false positive rate as the horizontal axis and the true positive rate as the vertical axis, and the area under it (AUC value) was used to measure the accuracy of the model. The range of AUC values was 0~1. When the AUC value was closer to 1, the false positive rate was smaller and the simulation effect was better. When it was greater than 0.9, the accuracy was very high, and 0.8–0.9 was good, 0.7–0.8 was average, 0.6–0.7 was poor, and 0.5–0.6 was failure [52]. The mean result was then imported into ArcGIS 10.8 software and divided into four parts according to the suitability rate, namely a non-suitable area (0–0.25), low-suitability area (0.25–0.50), medium-suitability area (0.50–0.75), and high-suitability area (0.75–1), for further mapping and area calculation. In addition, we assessed the relative importance of environmental variables using the percentage contribution and permutation importance generated by Maxent, complemented by a jackknife test to evaluate the relative contribution of each variable [53].

### 4.4. Centroid Migration Analysis of Suitable Habitats Across Time Periods

Utilizing ArcGIS, the potential suitable habitat of *C. marginatum* was treated as a continuous spatial unit and compressed into a single vector centroid point. This method provides a simplified geometric representation for analyzing spatiotemporal trends in habitat distribution. Centroid locations were derived for the mid-Holocene, current climate, and future periods under both SSP126 and SSP585 scenarios (2050s and 2070s). The position and directional shift in this geometric center reflect the integrated spatial distribution pattern and migration trajectory of *C. marginatum*’s suitable habitat across different climate periods, serving as a key indicator for identifying persistent climate refugia.

## 5. Conclusions

We compiled the geographical distribution data of *C. marginatum* and employed the Maxent model to simulate its potential suitable habitats, integrating multi-temporal climate data from the mid-Holocene, present, and future periods (2050s, 2070s), alongside terrain variables (elevation, slope, aspect) and soil properties. The results showed that precipitation (particularly precipitation of the driest month and annual precipitation) was the main environmental variable dominating the geographical distribution of *C. marginatum*. The current total area of the suitable habitat was approximately 98.38 × 10^4^ km^2^, mainly distributed in East China, Central China, Guangdong, and Guangxi. The high-suitability habitat was primarily located in the southern part of Hainan Island and the central–eastern part of Taiwan Island, with a scattered distribution also identified in northeastern Guangxi. From the mid-Holocene to the present, suitable habitats significantly expanded in regions including northern Jiangxi, southwestern Hubei, and the border areas of Hubei, Hunan, Guizhou, and Chongqing. However, under future climate scenarios (SSP126 and SSP585), the total suitable habitat area exhibited an overall contraction trend compared to the present (decreasing by 12.21% to 16.47%), and the area of the highly suitable habitat generally decreased (reducing by 19.75% to 44.44%). Crucially, northeastern Guangxi, Taiwan, and southern Hainan were identified as key climate refugia, maintaining relatively stable highly suitable habitats across all periods. This study offers recommendations for the preservation and utilization of *C. marginatum* germplasm resources and provides a scientific foundation for developing adaptation and protection plans, particularly prioritizing the conservation of the identified refugia in response to future climate change.

## Figures and Tables

**Table 1:** Contribution rates and permutation importance of the environmental factors for *C. marginatum*.

Variables	Percent Contribution/%	Permutation Importance/%
bio14	58.3	26.9
bio12	14.9	10.4
bio7	5.5	21.4
ALUM_SAT	4.9	1.3
DEM	2.4	13.4
bio4	2.3	1.3
CEC_CLAY	1.9	2.1
BSAT	1.7	1.8
bio15	1.6	4.9
bio2	1.1	1.9
SAND	0.8	0.4
Aspect	0.6	0.8
bio18	0.6	2.9
TCARBON_EQ	0.4	0.5
CLAY	0.4	0
CEC_EFF	0.4	0.1
GYPSUM	0.4	2.3
DRAINAGE	0.4	0.3
COARSE	0.4	2.2
BULK	0.3	0.1
SILT	0.3	0.7
bio8	0.2	0.1
TOTAL_N	0.2	0
ESP	0.1	0.1

**Table 2:** The suitable habitat area of *C. marginatum* (unit: 10^4^ km^2^).

Climate Scenario		Non-Suitable Area	Ratio/%	Low Suitability Area	Ratio/%	Medium-Suitability Area	Ratio/%	High Suitability Area	Ratio/%	Total Suitable Area	Ratio/%
Current		822.27		65.72		31.04		1.62		98.38	
Mid-Holocene		831.22	1.09	62.05	−5.58	26.65	−14.14	0.67	−58.64	89.37	−9.16
SSP126	2050s	836.37	1.71	63.52	−3.35	19.71	−36.50	1.05	−35.19	84.28	−14.33
	2070s	834.28	1.46	67.65	2.94	17.82	−42.59	0.90	−44.44	86.37	−12.21
SSP585	2050s	834.63	1.50	66.68	1.46	18.03	−41.91	1.30	−19.75	86.01	−12.57
	2070s	838.47	1.97	62.94	−4.23	18.11	−41.66	1.13	−30.25	82.18	−16.47

## Data Availability

The authors confirm that the data supporting the findings of this study are available within the article.

## Appendix A

**Table A1:** The environmental variables selected for this study.

Data Type	Environmental Variables	Description	Unit
Bioclimatic variables	Bio1	Annual Mean Temperature	°C
	Bio2	Mean Diurnal Range	°C
	Bio3	Isothermality	-
	Bio4	Temperature Seasonality	-
	Bio5	Max Temperature of Warmest Month	°C
	Bio6	Min Temperature of Coldest Month	°C
	Bio7	Temperature Annual Range	°C
	Bio8	Mean Temperature of Wettest Quarter	°C
	Bio9	Mean Temperature of Driest Quarter	°C
	Bio10	Mean Temperature of Warmest Quarter	°C
	Bio11	Mean Temperature of Coldest Quarter	°C
	Bio12	Annual Precipitation	mm
	Bio13	Precipitation of Wettest Month	mm
	Bio14	Precipitation of Driest Month	mm
	Bio15	Precipitation Seasonality	-
	Bio16	Precipitation of Wettest Quarter	mm
	Bio17	Precipitation of Driest Quarter	mm
	Bio18	Precipitation of Warmest Quarter	mm
	Bio19	Precipitation of Coldest Quarter	mm
Soil variables	ALUM_SAT	Aluminum Saturation	% ECEC
	BSAT	Base Saturation	%CECsoil
	BULK	Bulk Density	g/cm^3^
	CEC_CLAY	Clay Cation Exchange Capacity	cmolc/kg
	CEC_EFF	ECEC	cmolc/kg
	CEC_SOIL	CEC Soil	cmolc/kg
	CLAY	Clay	% weight
	CN_RATIO	Carbon/Nitrogen Ratio	(C/N)
	COARSE	Coarse Fragments	% volume
	DRAINAGE	Drainage	-
	ELEC_COND	Electric Conductivity	dS/m
	ESP	Exchangeable Sodium Percentage	%
	GYPSUM	Gypsum Content	% weight
	ORG_CARBON	Organic Carbon Content	% weight
	PH_WATER	pH in Water	−LOG(H^+^)
	REF_BULK	Reference Bulk Density	g/cm^3^
	SAND	Sand	% weight
	SILT	Silt	% weight
	TCARBON_EQ	Calcium Carbonate	% weight
	TEB	TEB	cmolc/kg
	TOTAL_N	Total Nitrogen Content	g/kg
Landform variables	DEM	Elevation	m
	Slope	Degree of Slope	°
	Aspect	Direction of Slope	-
